# Supplementary material for: Microbial metagenome-assembled genomes of the Fram Strait from short and long read sequencing platforms
Source: PeerJ. 2021 Jun 30;9:e11721. doi: 10.7717/peerj.11721 (PMC8254474; doi:10.7717/peerj.11721)
Supplement: Supplemental Information 2 — Upper four plots are example outputs of FastQC that summarises the per base sequence quality of Illumina reads. The bottom two plots are example outputs of NanoPlot that summarises the average read quality for different read lengths. [file peerj-09-11721-s002.pdf]

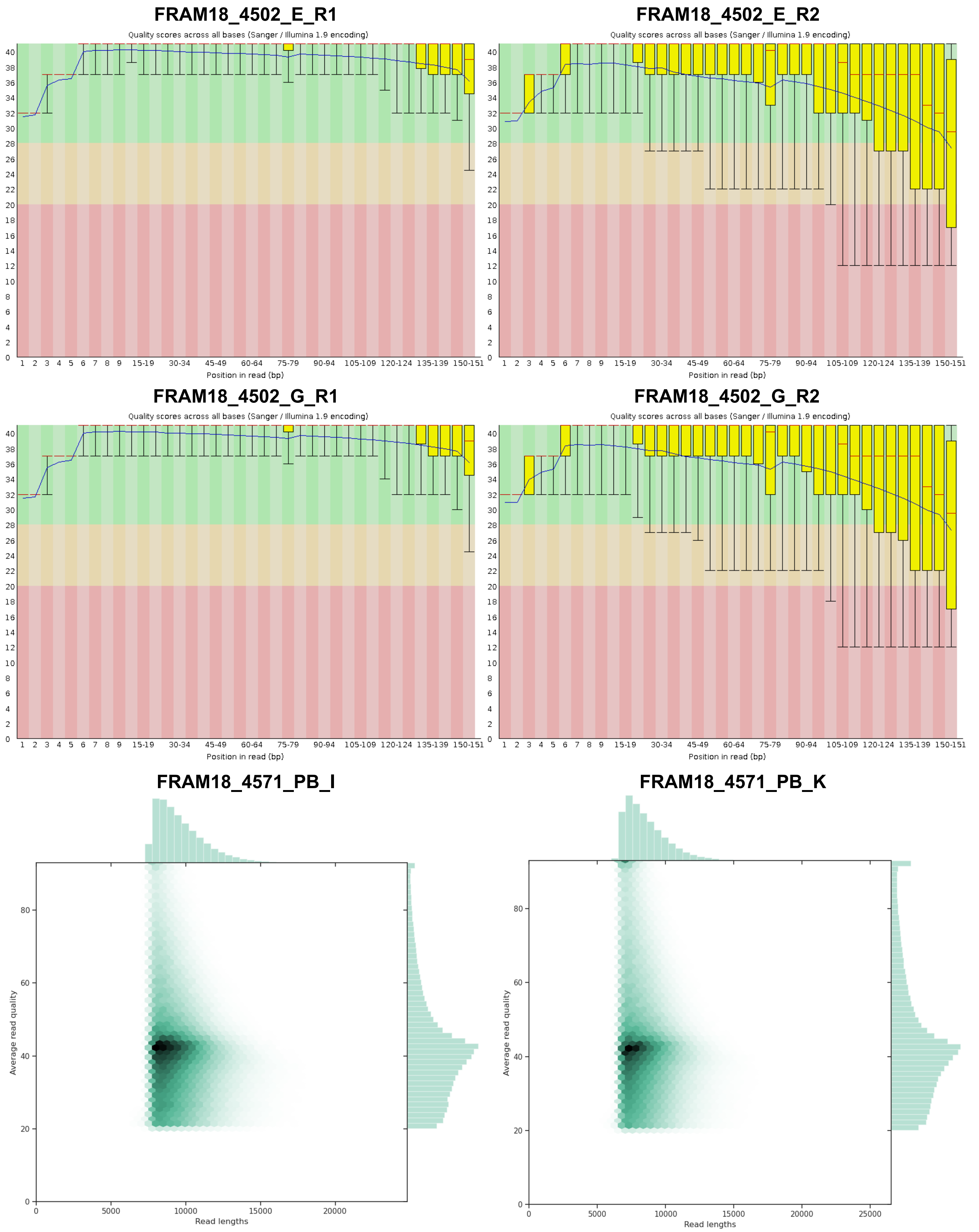

Supplementary Figure S1. Visualisation of sequence over length. Upper four plots are example outputs of FastQC that summarises the per base sequence quality of Illumina reads. The bottom two plots are example outputs of NanoPlot that summarises the average read quality for different read lengths.
