# Supplementary material for: Microbial metagenome-assembled genomes of the Fram Strait from short and long read sequencing platforms
Source: PeerJ. 2021 Jun 30;9:e11721. doi: 10.7717/peerj.11721 (PMC8254474; doi:10.7717/peerj.11721)
Supplement: Supplemental Information 3 — (A) Dendogram generated from Bray–Curtis dissimilarity matrix of samples’ community composition at a genus level, (B) Non-metric multi-dimensional scaling ordination of Bray–Curtis dissimilarity of samples’ community composition at a genus level. [file peerj-09-11721-s003.pdf]

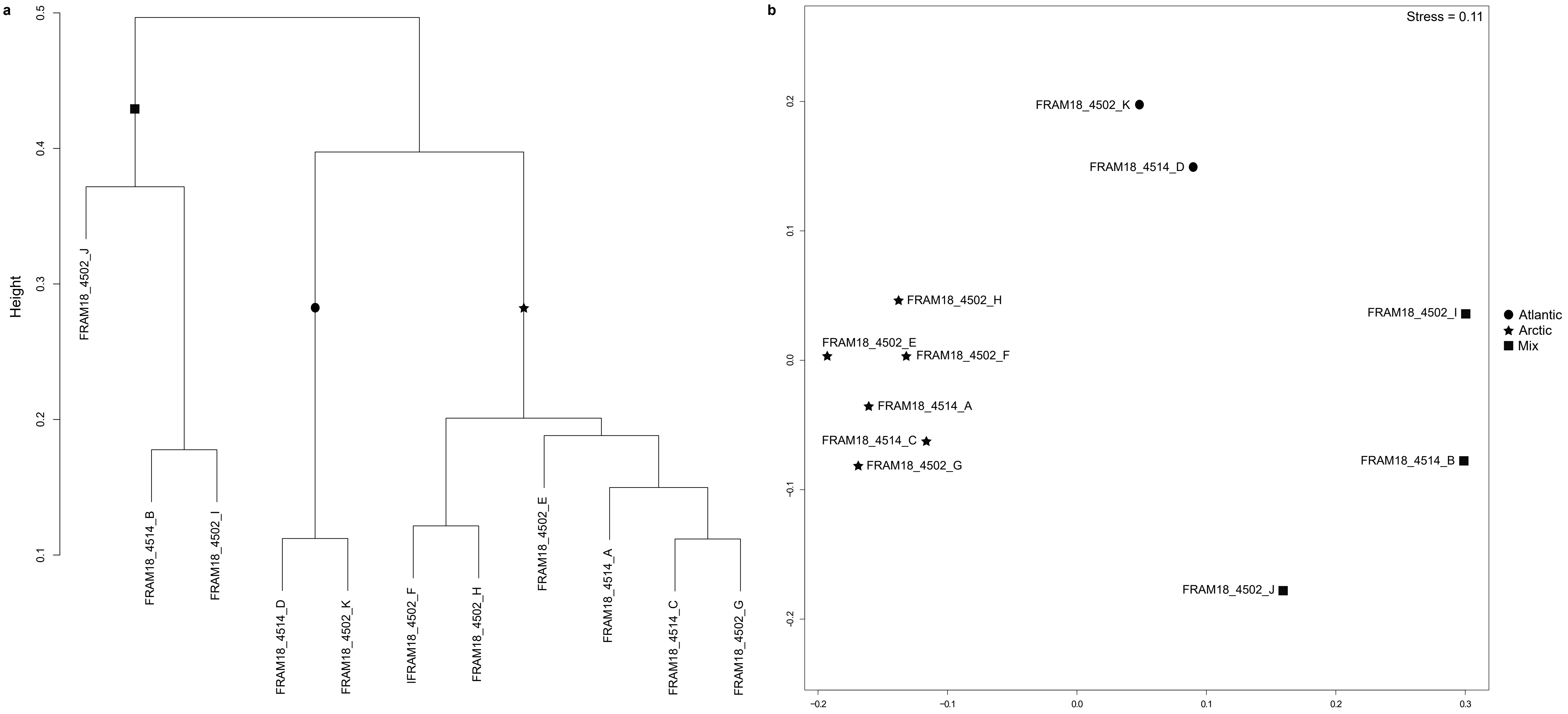

Supplementary Figure S2. Comparison of metagenomic samples based on Bray-Curtis dissimilarity of 16S rRNA gene composition. a) Dendogram generated from Bray-Curtis dissimilarity matrix of samples' community composition at a genus level, **b)** Non-metric multi-dimensional scaling ordination of Bray-Curtis dissimilarity of samples' community composition at a genus level.
